# Supplementary material for: Prophylactic Glatiramer Acetate Treatment Positively Attenuates Spontaneous Opticospinal Encephalomyelitis
Source: Cells. 2023 Feb 8;12(4):542. doi: 10.3390/cells12040542 (PMC9954767; doi:10.3390/cells12040542)
Supplement: Supplementary file 1 [file cells-12-00542-s001.zip › cells-2175699-supplementary.pdf]

SUPPLEMENT

Prophylactic Glatiramer Acetate Treatment positively attenuates Spontaneous Opticospinal Encephalomyelitis

Ümmügülsüm Koc [1], Steffen Haupeltshofer [1], Katharina Klöster [1], Seray Demir [1], Ralf Gold [1], Simon Faissner [1]

1 Department of Neurology, Ruhr-University Bochum, St. Josef-Hospital, Gudrunstr. 56, 44791 Bochum, Germany

Corresponding author

Simon Faissner, MD

Assistant Professor

Department of Neurology

St. Josef-Hospital, Ruhr-University Bochum

Gudrunstr. 56, 44791 Bochum, Germany

Tel: +49-234-5092411; Fax: +49-234-5092414

Email: simon.faissner@rub.de

SUPPLEMENTARY TABLES

Table S1: Fluorescent-labeled antibodies against cell surface proteins used for flow cytometry for cell surface protein detection.

Target	Label	Dilution	Company
CD4	APC	1:200	eBioscience 17-0042-82
CD8	FITC	1:200	BioLegend 100705
CD11b	FITC	1:200	BD Pharmingen 553310
CD11c	APC	1:200	eBioscience 17-0114-81
CD19	FITC	1:200	BD Pharmingen 553785
CD25	APC	1:200	eBioscience 17-0251-81A
CD45R/B220	APC	1:200	BD Biosciences 553086
CD69	PE	1:200	eBioscience 12-0691-81
MHCII	PE	1:200	BioLegend 107607

Table S2: Fluorescent-labeled antibodies against intracellular proteins.

Target	Label	Dilution	Company
Fox P3	PE	1:200	eBioscience 12-5773-82
IL-17A	PE	1:200	BD Pharmingen 559502
IFN-γ	FITC	1:200	eBioscience 11-7311-81

Table S3: Primary and secondary anti-mouse antibodies for immunofluorescent analysis.

First Antibody	Second Antibody
Anti-Iba1, polyclonal rabbit (1:500; Wako 019-18741)	Cy5 Anti-rabbit (1:1000; Abcam ab97077)
Anti-F4/80, monoclonal rat (1:300; BioRad MCA497GA)	Alexa 488 Anti-rat (1:1000; Thermo Scientific A10525)
Anti-CD11c Armenian Hamster (1:200; Abcam ab33483)	Alexa 488 Anti-Armenian Hamster (1:1000; Abcam ab173003)
